# Deep Learning-Based Prediction Model for Breast Cancer Recurrence Using Adjuvant Breast Cancer Cohort in Tertiary Cancer Center Registry

**DOI:** 10.3389/fonc.2021.596364

**Published:** 2021-05-04

**Authors:** Ji-Yeon Kim, Yong Seok Lee, Jonghan Yu, Youngmin Park, Se Kyung Lee, Minyoung Lee, Jeong Eon Lee, Seok Won Kim, Seok Jin Nam, Yeon Hee Park, Jin Seok Ahn, Mira Kang, Young-Hyuck Im

**Affiliations:** ^1^Division of Hematology-Oncology, Department of Medicine, Samsung Medical Center, Sungkyunkwan University School of Medicine, Seoul, South Korea; ^2^Digital Health Business Team, Samsung SDS, Seoul, South Korea; ^3^Department of Surgery, Samsung Medical Center, Sungkyunkwan University School of Medicine, Seoul, South Korea; ^4^Center for Health Promotion, Samsung Medical Center, Sungkyunkwan University School of Medicine, Seoul, South Korea

**Keywords:** breast cancer, surveillance, machine learning, recurrence model, real time prediction, adjuvant cohort

## Abstract

Several prognosis prediction models have been developed for breast cancer (BC) patients with curative surgery, but there is still an unmet need to precisely determine BC prognosis for individual BC patients in real time. This is a retrospectively collected data analysis from adjuvant BC registry at Samsung Medical Center between January 2000 and December 2016. The initial data set contained 325 clinical data elements: baseline characteristics with demographics, clinical and pathologic information, and follow-up clinical information including laboratory and imaging data during surveillance. Weibull Time To Event Recurrent Neural Network (WTTE-RNN) by Martinsson was implemented for machine learning. We searched for the optimal window size as time-stamped inputs. To develop the prediction model, data from 13,117 patients were split into training (60%), validation (20%), and test (20%) sets. The median follow-up duration was 4.7 years and the median number of visits was 8.4. We identified 32 features related to BC recurrence and considered them in further analyses. Performance at a point of statistics was calculated using Harrell's C-index and area under the curve (AUC) at each 2-, 5-, and 7-year points. After 200 training epochs with a batch size of 100, the C-index reached 0.92 for the training data set and 0.89 for the validation and test data sets. The AUC values were 0.90 at 2-year point, 0.91 at 5-year point, and 0.91 at 7-year point. The deep learning-based final model outperformed three other machine learning-based models. In terms of pathologic characteristics, the median absolute error (MAE) and weighted mean absolute error (wMAE) showed great results of as little as 3.5%. This BC prognosis model to determine the probability of BC recurrence in real time was developed using information from the time of BC diagnosis and the follow-up period in RNN machine learning model.

## Introduction

Breast cancer (BC) is the most common cancer affecting women worldwide and the most frequent cause of cancer death in women (1, 2). Recent advances in treatment strategies have improved BC-related mortality and morbidity; however, almost 30% of BC patients show recurrence in the follow-up. Therefore, to improve BC outcomes, it is necessary to focus on research such as improving screening methods for early detection of recurrence according to risk stratification, identifying new biomarkers, and developing new innovative treatment strategies.

There is an urgent unmet need to identify innovative methods to determine the prognosis of individual patients. Traditionally, clinicopathologic characteristics such as tumor size, axillary nodal status, histologic and nuclear grade, hormone receptors [estrogen receptor (ER) and progesterone receptor (PR)], and human epidermal growth factor receptor 2(HER2) status have been used to identify risk groups and to predict patient prognoses (3, 4). In addition to clinicopathologic characteristics, multigene signature panels offer an additional benefit in predicting patient prognoses (5, 6).

Several models for predicting the survival of individual BC patients have been proposed. Adjuvant! Online and PREDICT® are the online tools that predict durations of overall survival (OS) and disease-free survival (DFS) based on clinicopathologic factors (7–9), and CancerMath® shows the cancer-related mortality and life expectancy of BC patients (10). Survival rates predicted by those tools are used to determine the benefit of adjuvant chemotherapy after surgery or as reference data for shared decisions with patients about multiple treatments and surveillance (11). Most prediction models predict OS or DFS after BC diagnosis or curative surgery. However, they did not reflect newly developed comorbidities and test results, which may affect BC-specific recurrence or death with time during surveillance (12, 13).

Using recent advances in various machine learning algorithms (14, 15), some researchers have worked to develop models that can consider a large amount of complex data, and many efforts are being made to more accurately predict the survival of individual BC patients. The attention-based multi-NMF DNN (AMND) model based on a deep neural network was proposed to predict the survival of BC with the gene expression profile and clinical data of 1,489 patients (16). The area under the curve (AUC) value of the AMND model was 87.04%. The rule-based trees random forest model (TRF) was developed for the prediction of BC survival with 900 patients (17). The classification performance of this method showed an AUC of 93%. In addition, there was breast cancer recurrence prediction based on SVM (BCRSVM) for BC recurrence prediction within 5 years after BC surgery with 679 patients. This model suggested an AUC of 85% for the proposed model with seven time-independent variables as the most informative way of predicting recurrence (18). Despite these successes, there is still no predictive tool for individual survival available to determine appropriate follow-up periods and test methods for individual patients who have completed curative surgery and adjuvant treatment (16, 17).

Therefore, this study developed a recurrence prediction model of individual BC patients using the machine learning method. This model was developed using BC-related clinicopathologic factors at the time of curative surgery and consecutive clinical factors that have been identified during the BC surveillance period.

## Methods

### Study Population

**Figure 1:**
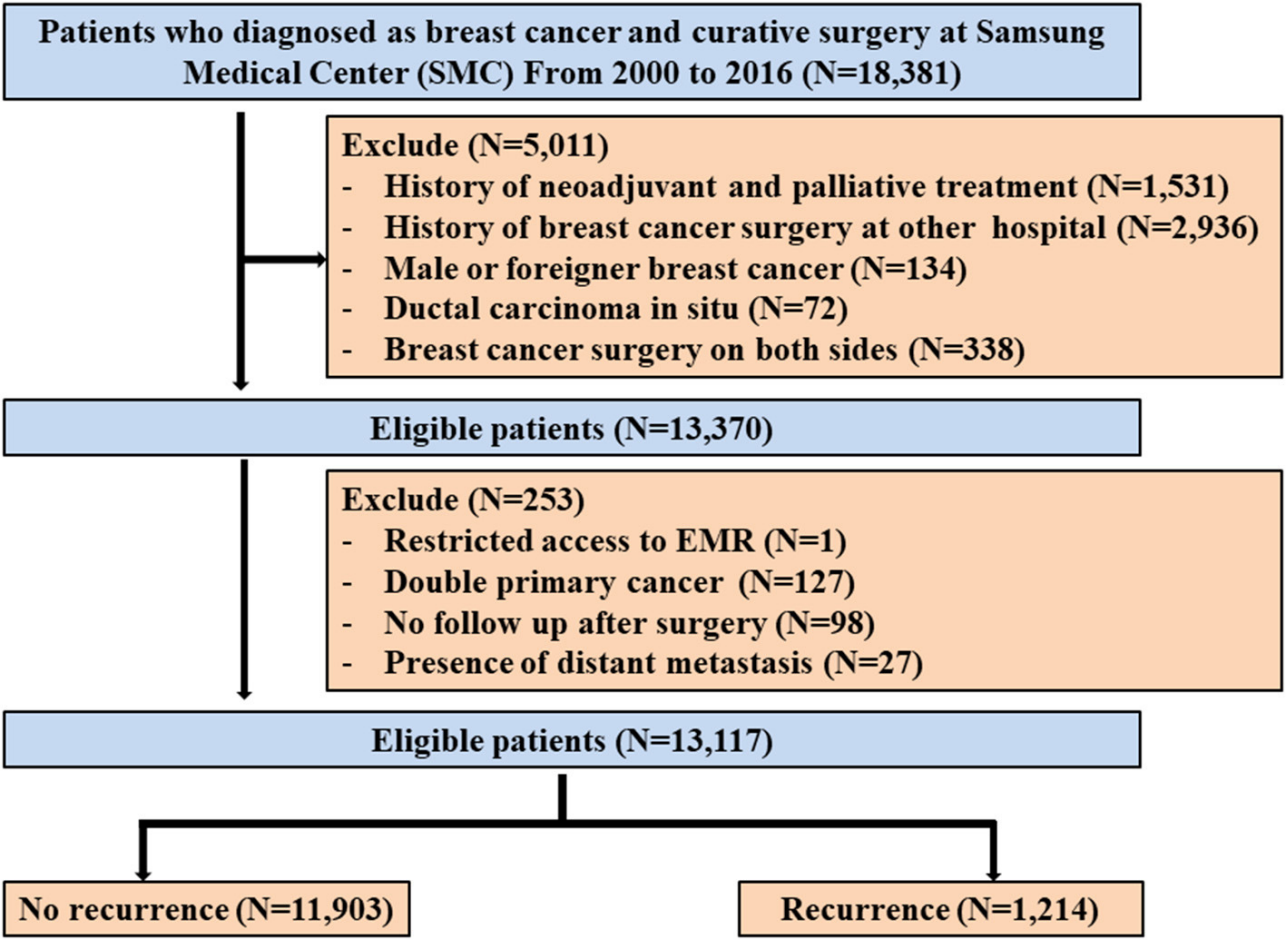
Study cohort.

This is a retrospective data analysis from the BC registry composed of BC patients who received curative surgery followed by adjuvant treatment including chemotherapy, radiotherapy, endocrine therapy, and targeted therapy at Samsung Medical Center between January 2000 and December 2016. Patients who received neoadjuvant chemotherapy before surgery were diagnosed with ductal carcinoma *in situ*, were male or foreigners, and have a history of BC surgery at another hospital were excluded. Among the remaining 13,370 patients in this registry, we also excluded 253 patients with any of the following conditions: (1) restricted access to electronic medical record (EMR) (*n* = 1), (2) double primary cancer (*n* = 127), (3) no follow-up after surgery (*n* = 98), or (4) presence of distant metastases (*n* = 27) (Figure 1). Therefore, we analyzed the data of 13,117 patients. This study was reviewed and approved by the Institutional Review Board (IRB) of Samsung Medical Center, Seoul, Korea (IRB No. 2018-06-137), with an informed consent waiver, due to the use of retrospective clinical data.

### Measurements

Detailed information on surgery, adjuvant chemotherapy, radiotherapy, endocrine therapy, and targeted therapy were obtained from EMR. The pathologic stage was based on the criteria of the American Joint Committee on Cancer, 7th Edition (4). Two experienced pathologists reviewed and determined the primary tumor characteristics based on size, axillary nodal status, and receptor status (ER, PR, and HER2) by immunohistochemical (IHC) staining. ER positivity and PR positivity were defined as an Allred score of 3–8 based on IHC staining with antibodies against ER (Immunotech, France) and PR (Novocastra, UK), respectively. HER2 status was evaluated using the appropriate antibody (Dako, CA) and/or silver *in situ* hybridization (SISH). HER2 grades 0 and 1 indicated a negative result, while grade 3 indicated a positive result. Amplification of HER2 was confirmed by SISH for results of 2+. Triple negative BC was defined as BC with negative ER and PR expression, and lack of HER2 overexpression. In terms of radiologic tests, the categories of mammography and breast sonography were reported according to BI-RADS® (Breast Imaging, Reporting & Data System) (19), which is a risk assessment and quality assurance tool developed by the American College of Radiology. In this study, recurrence was defined as the first detected event of local and/or distant BC recurrence.

### Data Pre-processing

The initial data set contained 325 clinical data elements including baseline characteristics with patient demographics, clinical information, and laboratory test results at the time of BC diagnosis, pathologic information including tumor size, nodal status, histologic characteristics, and IHC information for ER, PR, HER2, Ki-67, cytokeratin 5/6 (CK5/6), epidermal growth factor receptor(EGFR), and follow-up clinical information including laboratory and imaging data during surveillance (Supplementary Table 1).

Characteristic values including the subtype and stage were transformed into nominal or ordinal numeric values. For continuous variables, log transformation was used to deal with skewed data as needed, and Z-score normalization was applied. To reduce the number of discrete intervals for a continuous attribute, data binning divided continuous features (Ki-67) into a pre-specified number of categories (25% or 10% units), thereby making the data discrete. Categorical variables were one-hot encoded for the data analysis. For missing data, we used the average method for the data at the first time point and the last observation carried forward (LOCF) method for the data at a later time point.

### Feature Selection

Potential independent variables selected by univariate analyses were considered in a time-dependent Cox regression model. A backward selection procedure applying the Akaike information criterion (AIC) (20) was used to select the final multivariable model. The backward stepwise selection procedure began with a model that included potential independent variables, then, the least significant variable was removed, and the model was run again based on AIC, in a stepwise manner until there were no variables left to remove. All analyses were performed using the R v3.6 software, and the significance level was set at 0.05. A few variables not significant at the 0.05 level were manually selected from the clinical point of view for the final multivariable model.

### Deep Learning-Based Survival Algorithm

Before constructing the deep learning models, we randomly created 60/20/20 mutually exclusive sets for training, validation, and testing while preserving the same proportion of recurrence events in all three sets.

Weibull Time To Event Recurrent Neural Network (WTTE-RNN) by Martinsson was implemented as an open-source Python module (https://github.com/ragulpr/wtte-rnn) (21). The deep survival model took data about each patient's time-independent features (age at operation, molecular tests results, hormone receptor stage, pathologic stage, etc.) and time-dependent features (i.e., lab test results, mammography, etc.) as input. The system represented those inputs as matrix A of size *m* × *n* × *k*, where *m* is the total number of follow-ups in patient records, *n* is the window size of the follow-ups, and *k* is the total number of features. We did a grid search to find the optimal window size as time-stamped inputs. The network contained 32 cells at the first hidden layer and 20 cells at the second layer. We used hyperbolic tangent activation layers after the recurrent layers with gated recurrent units that take the time domain into account. Adam was used as the optimizer with an initial learning rate of 0.001, which was reduced by a factor of 10 when the model stopped improving after iterations. The model was trained with the batch size set to 100. The dropout rate was set to 0.25. The network structure was implemented in Python, using Keras with a Tensorflow backend (Python 3.5, Keras 2.1.2, Tensorflow 1.4.0).

To compare our model with existing models, we also developed logistic regression, random forest, and gradient boosting machine learning models because they are the methods most typically used in medical applications. The optimal configuration for the hyperparameters of each machine learning model was set by testing a wide range of parameters in a grid search.

### Performance Evaluation

We used Harrell's concordance-index (C-index) in the lifeline package in Python to measure the concordance between the predicted recurrence time and the actual recurrence time (22, 23).

The AUC was used to assess the 2-, 5-, and 7-year recurrence predictions. To evaluate the model performance based on the main BC features, we stratified patients by their pathologic T stage, pathologic N stage, hormone receptor, and HER2 (ER/PR/HER2) status, and EGFR and CK5/6 status. The predicted recurrence values calculated by our model were compared with the actual recurrence in each group over 2, 5, and 7 years. Model ‘specificity and accuracy were assessed using the Median Absolute Error (MAE), mean absolute error, weighted Mean Absolute Error (wMAE), and maximum error.

Data sets for which the follow-up period was shorter than the prediction period and no recurrence occurred were excluded from the performance evaluation.

## Results

### Clinical Characteristics

The baseline characteristics of the patients are summarized in Table 1. The median follow-up was 4.7 years (interquartile range: 3.0–7.7 years). Of the 13,117 patients in the study population, BC recurrence occurred in 1,214 (9.2%) patients during the follow-up period. The median age at BC curative surgery was 48 years (interquartile range, 43–55), and patients who did not experience BC recurrence were slightly older than those who did (median age: 48 vs. 46, *p* < 0.001). The proportion of BC subtypes differed between patients with and without BC recurrence. Hormone receptor (HR)+, defined as ER and/or PR+, HER2– BCs were more frequently observed in patients without recurrence (64.7 vs. 50.8%, *p* < 0.001), whereas ER-HER2– BCs were less frequent in patients without recurrence (12.5 vs. 22.2%, *p* < 0.001). Pathologic stage also affected BC recurrence: higher T (T3 and T4) and N (N2 and N3) stages were more frequently observed in patients with BC recurrence (p < 0.001 for all).

**Table 1:** Characteristics of the study population.

**Variable**	**Total *N* = 13,117**	**Disease free *N* = 11,903**	**Recurrent *N* = 1,214**	***P*-value**
**Age**				
Mean ± SD	49.2 ± 9.9	49.4 ± 9.9	46.7 ± 10.5	<0.001
Median (interquartile range)	48.0 [43.0; 55.0]	48.0 [43.0; 55.0]	46.0 [39.0; 53.0]	<0.001
**Menopausal status**				0.001
Pre-menopausal	7,576 (57.8%)	6,816 (57.3%)	760 (62.6%)	
Post-menopausal	5,481 (41.8%)	5,033 (42.3%)	448 (36.9%)	
Unknown	60 (0.5%)	54 (0.5%)	6 (0.5%)	
**BMI (kg/m**^**2**^**)**				0.783
Underweight	455 (3.5%)	414 (3.5%)	41 (3.4%)	
Normal	6,011 (45.8%)	5,450 (45.8%)	561 (46.2%)	
Overweight	3,032 (23.1%)	2,756 (23.2%)	276 (22.7%)	
Obesity	3,136 (23.9%)	2,852 (24.0%)	284 (23.4%)	
High obesity	477 (3.6%)	425 (3.6%)	52 (4.3%)	
Unknown	6 (0.0%)	6 (0.1%)	0 (0.0%)	
**Bilateral breast cancer**				0.999
Yes	13 (0.1%)	12 (0.1%)	1 (0.1%)	
No	13,104 (99.9%)	11,891 (99.9%)	1,213 (99.9%)	
Baseline CA 15-3 (Mean ± SD)	10.5 ± 11.2	10.4 ± 10.8	12.5 ± 14.2	<0.001
**Pathologic T stage**				<0.001
T1	7,947 (60.6%)	7,415 (62.3%)	532 (43.8%)	
T2	4,594 (35.0%)	4,026 (33.8%)	568 (46.8%)	
T3	509 (3.9%)	417 (3.5%)	92 (7.6%)	
T4	17 (0.1%)	11 (0.1%)	6 (0.5%)	
Unknown	50 (0.4%)	34 (0.3%)	16 (1.3%)	
**Pathologic** ***N*** **stage**				<0.001
N0	8,304 (63.3%)	7,756 (65.2%)	548 (45.1%)	
N1	3,390 (25.8%)	3,044 (25.6%)	346 (28.5%)	
N2	871 (6.6%)	715 (6.0%)	156 (12.9%)	
N3	487 (3.7%)	335 (2.8%)	152 (12.5%)	
Unknown	65 (0.5%)	53 (0.4%)	12 (1.0%)	
**Histologic grade**				<0.001
Well	3,186 (24.3%)	3,067 (25.8%)	119 (9.8%)	
Moderate	5,431 (41.4%)	4,949 (41.6%)	482 (39.7%)	
Poorly	4,036 (30.8%)	3,490 (29.3%)	546 (45.0%)	
Unknown	464 (3.5%)	397 (3.3%)	67 (5.5%)	
**Lymphovascular invasion**				<0.001
Yes	3,846 (29.3%)	3,315 (27.9%)	531 (43.7%)	
No	8,749 (66.7%)	8,147 (68.4%)	602 (49.6%)	
Unknown	522 (4.0%)	441 (3.7%)	81 (6.7%)	
**Nipple areolar complex involvement**				<0.001
Yes	1,279 (9.8%)	1,142 (9.6%)	137 (11.3%)	
No	7,682 (58.6%)	6,836 (57.4%)	846 (69.7%)	
Unknown	4,156 (31.7%)	3,925 (33.0%)	231 (19.0%)	
**Estrogen receptor**				<0.001
Positive	9,853 (75.1%)	9,083 (76.3%)	770 (63.4%)	
Negative	3,219 (24.5%)	2,784 (23.4%)	435 (35.8%)	
Unknown	45 (0.3%)	36 (0.3%)	9 (0.7%)	
**Progesterone receptor**				<0.001
Positive	8,981 (68.5%)	8,302 (69.7%)	679 (55.9%)	
Negative	4,089 (31.2%)	3,563 (29.9%)	526 (43.3%)	
Unknown	47 (0.4%)	38 (0.3%)	9 (0.7%)	
**HER2**				0.005
Positive	2,729 (20.8%)	2,434 (20.4%)	295 (24.3%)	
Negative	10,079 (76.8%)	9,192 (77.2%)	887 (73.1%)	
Unknown	309 (2.4%)	277 (2.3%)	32 (2.6%)	
**Subtype**				<0.001
ER or PR+, HER2–	8,321 (63.4%)	7,704 (64.7%)	617 (50.8%)	
ER or PR+, HER2+	1,471 (11.2%)	1,310 (11.0%)	161 (13.3%)	
ER and PR–, HER2-	1,758 (13.4%)	1,488 (12.5%)	270 (22.2%)	
ER and PR–, HER2+	1,258 (9.6%)	1,124 (9.4%)	134 (11.0%)	
Unknown	309 (2.4%)	277 (2.3%)	32 (2.6%)	
**CK5/6**				<0.001
Positive	1,571 (12.0%)	1,380 (11.6%)	191 (15.7%)	
Negative	8,633 (65.8%)	8,150 (68.5%)	483 (39.8%)	
Unknown	2,913 (22.2%)	2,373 (19.9%)	540 (44.5%)	
**EGFR**				<0.001
Positive	2,090 (15.9%)	1,850 (15.5%)	240 (19.8%)	
Negative	8,111 (61.8%)	7,677 (64.5%)	434 (35.7%)	
Unknown	2,916 (22.2%)	2,376 (20.0%)	540 (44.5%)	
**Ki67, %**				<0.001
0–9	2,344 (17.9%)	2,252 (18.9%)	92 (7.6%)	
10–19	2,057 (15.7%)	1,912 (16.1%)	145 (11.9%)	
20–29	1,263 (9.6%)	1,146 (9.6%)	117 (9.6%)	
30–39	956 (7.3%)	853 (7.2%)	103 (8.5%)	
40–49	446 (3.4%)	399 (3.4%)	47 (3.9%)	
50–59	489 (3.7%)	419 (3.5%)	70 (5.8%)	
60–69	422 (3.2%)	370 (3.1%)	52 (4.3%)	
70–79	285 (2.2%)	251 (2.1%)	34 (2.8%)	
80–89	321 (2.4%)	276 (2.3%)	45 (3.7%)	
90–99	190 (1.4%)	158 (1.3%)	32 (2.6%)	
Unknown	4,344 (33.1%)	3,867 (32.5%)	477 (39.3%)	
**Radiotherapy**				<0.001
Yes	9,496 (72.4%)	8,684 (73.0%)	812 (66.9%)	
No	3,427 (26.1%)	3,043 (25.6%)	384 (31.6%)	
Stop by patient	2 (0.0%)	1 (0.0%)	1 (0.1%)	
Unknown	192 (1.5%)	175 (1.5%)	17 (1.4%)	
**Chemotherapy**				<0.001
Yes	8,572 (65.4%)	7,596 (63.8%)	976 (80.4%)	
No	4,367 (33.3%)	4,141 (34.8%)	226 (18.6%)	
Stop by patient	36 (0.3%)	32 (0.3%)	4 (0.3%)	
Unknown	142 (1.1%)	134 (1.1%)	8 (0.7%)	
**Hormone therapy**				<0.001
Yes	9,747 (74.3%)	8,995 (75.6%)	752 (61.9%)	
No	3,162 (24.1%)	2,717 (22.8%)	445 (36.7%)	
Unknown	208 (1.6%)	191 (1.6%)	17 (1.4%)	
**Targeted therapy**				<0.001
Yes	1,446 (17.9%)	1,333 (18.9%)	113 (10.8%)	
No	6,303 (77.8%)	5,409 (76.7%)	894 (85.1%)	
Unknown	349 (4.3%)	306 (4.3%)	43 (4.1%)	

*For comparison of non-recurrent vs. recurrent patients.

*ER, estrogen receptor; HER2, human epidermal growth factor receptor 2; PR, progesterone receptor; EGFR, epidermal growth factor receptor; SD, standard deviation*.

**Figure 2:**
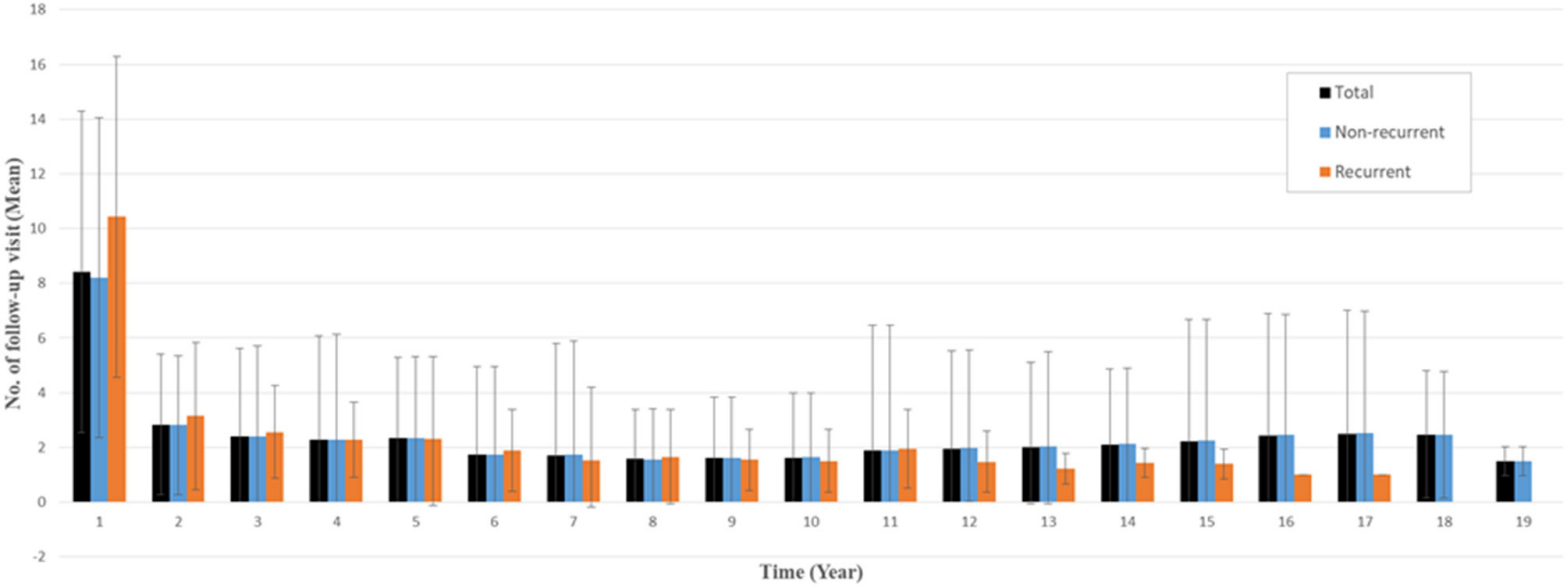
Mean numbers of follow-up visits per year after surgery. Error bar indicates standard deviation.

The number of follow-up visits was measured from the date of surgery to the last follow-up, including all-cause mortality. Overall, the mean number of follow-up visits within 1 year after surgery was 8.4 for all patients (10.4 for recurrent patients and 8.2 for nonrecurrent patients), and the mean number of follow-up visits more than 1 year after surgery was <3 (Figure 2).

### Prognosis Feature Selection

Univariate/multivariate analyses and backward stepwise selection identified 27 prognostic features that affected BC recurrence. In addition, five features (synchronous contralateral cancer, adjuvant chemotherapy, serum alkaline phosphatase, serum alanine aminotransferase, and serum calcium) were included in the further analyses based on expert opinions. Therefore, we used 32 features to develop the BC recurrence model. The prognosis features used in the BC recurrence model were as follows: 13 features related to baseline clinicopathologic characteristics, four features about adjuvant treatments, and 15 follow-up (time-dependent) features, which were serial measurements taken during follow-up (Supplementary Table 1).

### Model Training and Performance

To learn the various measurement period data, we needed to find a fixed value for the window size (*k*) during the training stage. Longer periods are better, but a look-back period of 12 months was the optimal window size for our training data set, according to the results of grid-search algorithms.

**Figure 3:**
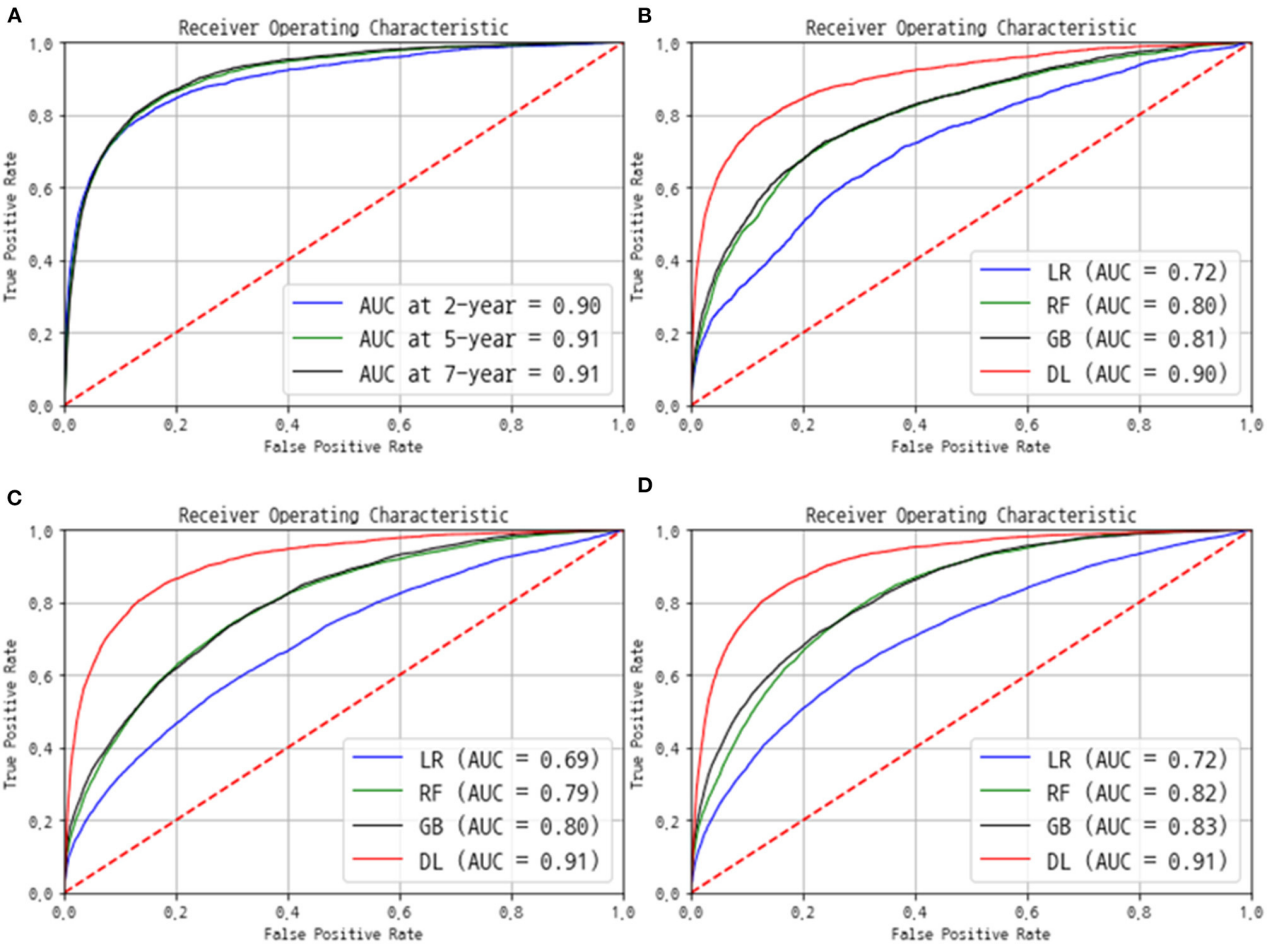
Performance of the final model in terms of the receiver operating characteristic (ROC) curves. **(A)** The ROC and area under the ROC curve (AUC) were evaluated using the separated test group at each 2-, 5-, and 7-year point. The comparison of ROC and AUC of logistic regression (LR), random forest (RF), gradient boosting (GB), and our deep-learning (DL) models at **(B)** 2 year, **(C)** 5 year, and **(D)** 7 year point.

The AUC and C-index were used to evaluate the performance. The C-index eventually reached 0.92 for the training data set and 0.89 for the validation and test data sets. The AUC value was 0.90 at the 2-year point, 0.91 at the 5-year point, and 0.91 at the 7-year point (Figure 3A). We also compared our model with the performances of three other machine learning prediction models (Figures 3B–D). The logistic regression model produced AUC in the range of 0.69–0.72, and the random forest and gradient boosting methods, which are the most popular ensemble models, showed similar AUC values in the range of 0.80–0.83. The deep learning-based final model only exceeded an AUC of 0.90, outperforming the existing machine learning-based models.

**Figure 4:**
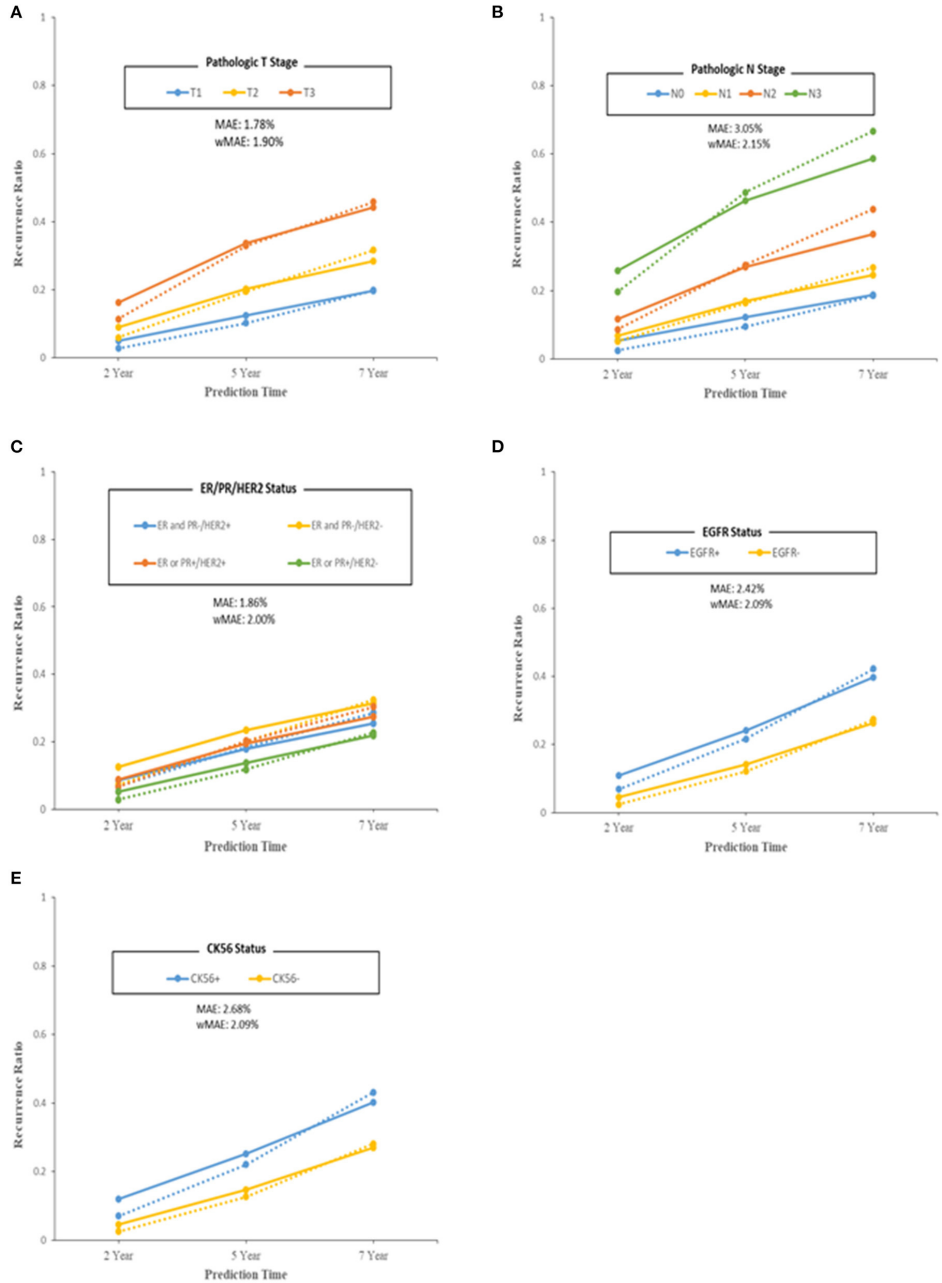
Performance of the final model in terms of model error (predicted–observed) over each of the 2/5/7 years. Patients were grouped by breast cancer features. **(A)** Pathologic T stage. **(B)** Pathologic N stage. **(C)** ER/PR/HER2 status. **(D)** EGFR status. **(E)** CK56 status. For model specificity and accuracy, Median Absolute Error (MAE) and weighted Mean Absolute Error (wMAE) were calculated at each group or bin. Solid lines indicate observed recurrence proportion, and dashed lines indicate predicted recurrence proportion.

We also evaluated the model from a clinical point of view with pathologic T stage, pathologic N stage, subtypes according to ER/PR and HER2 status, EGFR status, and CK5/6 status by comparing the predicted recurrence proportion with the actual recurrence proportion (Figure 4). The MAE and wMAE of each group showed great results of as little as 3.5%. The model errors for pathologic T stage and N stage features were similar to each other but differed from those for the other pathologic features. The subtypes had similar error values of around 2.5%. The discrimination of the wMAE at each prediction time (2, 5, and 7 years) showed only small differences (Supplementary Tables 2–6).

## Discussion

In this study, we developed an individually conditional BC recurrence prediction model using machine learning and an adjuvant BC cohort in a tertiary cancer hospital. We used baseline patient clinical characteristics, pathologic characteristics after curative surgery, and the results of follow-up tests, including laboratory tests, mammography, and breast sonography.

Machine learning is currently used for a wide range of applications in cancer research (24). Imaging diagnosis and pathologic diagnosis of BCs have been broadly supported by machine learning algorithms. A large scale retrospective analysis has indicated that artificial intelligence (AI) algorithms can improve BC detection ability on mammography better than radiologists can (25). In addition, AI has improved breast MRI interpretation (26) and predictions of the response to neoadjuvant chemotherapy (27). In terms of pathologic diagnosis, AI has helped pathologists to precisely diagnose BC using digital image analysis (28). Moreover, AI can interpret comprehensive genetic information to predict tumor site of origin (29).

Previous machine learning models of BC prognosis prediction were developed using baseline clinical and pathologic information (30–32). Most of those studies used pathologic information and additional molecular information, such as the intrinsic subtype at BC diagnosis. However, molecular information is not given in routine clinical practice.

Current surveillance studies suggested that BC recurrence was influenced by clinical information during the follow-up period as well as at the time of diagnosis. For example, alcohol consumption (33) and obesity at postmenopausal status (34) were well-known risk factors for BC recurrence. In terms of laboratory tests, changes in CA-15-3 during follow-up were traditionally used to detect BC recurrence (35, 36), and C-reactive protein was also considered as a predictive biomarker for BC recurrence (37). Moreover, surveillance guideline recommends annual mammography in BC patients after curative BC surgery (38). However, previous machine learning studies of the prediction model of BC prognosis have not considered follow-up exam data such as imaging and laboratory tests.

We used the same BC surveillance guideline since early 2010 (39, 40). Those guidelines recommend taking a careful history and performing a physical examination every 6–12 months, including regular mammography 6 months after the completion of definitive radiation therapy. In addition, the use of complete blood counts, chemistry panels, and tumor markers (CEA, CA-15-3) is not recommended for routine follow-up in an otherwise asymptomatic patient with no specific findings on clinical examination according to those guidelines. Understanding of the nature and biology of BC has improved, and it is now known that the timing and pattern of BC recurrence differ for patients with different BC subtypes (41). Moreover, the current concept of oligometastasis in BC, defined as low-volume metastatic disease with a few, small metastatic lesions, considered BC patients with oligometastasis to be a distinct subgroup with a more favorable long-term prognosis than patients with metastatic BC (42). This suggested that an early diagnosis of BC recurrence, rather than waiting for patients to show symptoms, might thus confer a survival benefit. Therefore, improved screening programs that incorporate the biology of individual BC patients and a method to precisely predict the risk of recurrence for individual patients are urgently needed.

For this study, we utilized an RNN model that weight features at BC diagnosis, treatment, and follow-up. In RNNs, the output of a hidden unit at the current time step is fed back into the hidden unit so that it forms part of the input for the previous time steps. This allows RNNs to fit and make predictions from sequences of events ordered chronologically. In addition, we applied the Weibull distribution instead of the well-known Cox regression model to our deep learning framework. The Weibull distribution allows more flexibility than other survival models because the associated hazard rate is not constant with respect to time, which helps to estimate the length of the hazard during the cancer recurrence period when using follow-up data.

In this model, the T and N stages and lymphovascular invasion at the time of curative surgery affected BC prognosis as stationary variables. In terms of IHC, neither ER, PR, nor HER2 affected BC prognosis. These three IHC components are used to categorize BC when choosing endocrine therapy, chemotherapy, and targeted therapy (38). Although these factors have been understood as important prognostic and predictive biomarkers for BC recurrence, proper treatment according to the BC subtype would neutralize their prognostic effects (43).

Our machine learning prognostic model uses baseline, treatment, and follow-up variables. In this analysis, we focused on laboratory tests during the follow-up period. An increase in the white blood cell count, hemoglobin, and total protein had a protective effect against BC recurrence, whereas elevated levels of serum glucose, absolute neutrophil count, and CA-15-3 increased the risk of BC recurrence (Supplementary Table 1). A previous study to find the relationship between BC prognosis and laboratory tests indicated that hemoglobin, alkaline phosphatase, and prothrombin time were associated with BC prognosis (44). In other cancer types, the association between the lymphocyte–monocyte ratio and cancer recurrence was studied (45, 46). However, those previous studies used the results from perioperative blood tests, not serial follow-up data.

In terms of follow-up imaging tests, the results of mammography and ultrasonography were naturally affected BC recurrence. Current studies with supplementary ultrasonography adding to mammography would help to detect BC recurrence but increase false-positive findings, and therefore, guidelines for BC surveillance did not recommend (47, 48).

The tests we used in our model were routinely performed at every follow-up visit. Thus, our machine learning model for BC prognosis was made with maximal use of the laboratory test results from current surveillance practices without requiring other laboratory work, such as intrinsic subtyping. Therefore, our BC prognosis model could fit into routine clinical practice better than previous machine learning models. Moreover, we can adapt this prognosis model into our EMRs using a website and thereby acquire information about BC recurrence in real time. This model could thus present the recurrence risk at each follow-up point using all available laboratory and imaging test results.

The results of this study should be interpreted in light of some limitations. Because our study was limited to a single institution, our results might not be generalizable to other cancer patients in other settings. Therefore, the findings from our study should be validated using samples from other institutions to confirm generalizability. Nonetheless, our model is the first machine learning-based BC prognosis model developed using clinical information at both BC diagnosis and follow-up. Moreover, our model produced high AUC scores that remained consistent for several years after the completion of BC treatment.

In conclusion, we used an RNN machine learning model and data from an adjuvant BC cohort in a tertiary cancer institute to develop a BC prognosis model that considers information from the time of BC diagnosis and during the follow-up period. This model can rapidly and precisely predict the probability of BC recurrence. A retrospective validation study using another adjuvant BC cohort and a prospective validation study are warranted.

## Data Availability Statement

The original contributions presented in the study are included in the article/Supplementary Material. Further inquires can be directed to the corresponding author.

## Ethics Statement

This study was reviewed and approved by the Institutional Review Board (IRB) of Samsung Medical Center, Seoul, Korea (IRB No. 2018-06-137). This study was performed in accordance with the Declaration of Helsinki. Written informed consent for participation was not required for this study in accordance with the national legislation and the institutional requirements.

## Author Contributions

Y-HI, MK, and YP conceived and planned the experiments. J-YK, YL, and JY carried out the analyses and experiments. J-YK, SL, ML, JL, SK, SN, YP, JA, and Y-HI contributed to the collection of the samples and clinical data. J-YK, YL, YP, ML, MK, and Y-HI contributed to the interpretation of the results. J-YK, YL, and JY took the lead in writing the manuscript. Y-HI supervised the project. All authors reviewed and confirmed manuscript.

## Conflict of Interest

YL, YP, and ML were employed by the company Samsung SDS. The remaining authors declare that the research was conducted in the absence of any commercial or financial relationships that could be construed as a potential conflict of interest.
