# Supplementary material for: Deep Learning-Based Prediction Model for Breast Cancer Recurrence Using Adjuvant Breast Cancer Cohort in Tertiary Cancer Center Registry
Source: Front Oncol. 2021 May 4;11:596364. doi: 10.3389/fonc.2021.596364 (PMC8129587; doi:10.3389/fonc.2021.596364)
Supplement: Supplementary file 1 [file Data_Sheet_1.docx]

**Supplementary Table 1.** Univariate and multivariate analysis of prognostic factors for prediction of breast cancer

| Variable | Univariate HR (95% CI) | Multivariable HR (95% CI) |
| --- | --- | --- |
| **Axillary surgery type** |  |  |
| SLNB | *Reference* | *Reference* |
| ALND | 2.37 (2.11, 2.66)** | 1.75 (1.42, 2.15)** |
| others | 2.90 (1.65, 5.07)** | 0.91 (0.31, 1.66) |
| **Synchronous contralateral cancer^b^** | 1.20 (0.81, 1.77) | 1.21 (0.84, 1.76) |
| **CA 15-3 (before operation) ^a^** | 1.00 (1.00, 1.01)** | 0.99 (0.97, 1.01) |
| **Pathologic T stage** |  |  |
| T1 | *Reference* | *Reference* |
| T2 | 1.85 (1.64, 2.08)** | 1.34 (1.17, 1.54)** |
| T3 | 2.94 (2.35, 3.67)** | 1.36 (1, 1.86)* |
| T4 | 4.63 (1.93, 11.08)** | 0.69 (0.14, 3.44) |
| Unknown | 8.95 (5.13, 15.62)** | 11.6 (2.33, 57.97) |
| **Pathologic N stage** |  |  |
| N0 | *Reference* | *Reference* |
| N1 | 1.48 (1.29, 1.69)** | 0.98 (0.81, 1.18) |
| N2 | 2.56 (2.15, 3.06)** | 1.42 (1.08, 1.88)* |
| N3 | 5.22 (4.35, 6.27)** | 1.72 (1.09, 2.71)* |
| Unknown | 4.03 (2.15, 7.54)** | 1.09 (0.26, 4.65) |
| **Lymphatic invasion categories** |  |  |
| Yes | *Reference* | *Reference* |
| No | 0.46 (0.41, 0.52)** | 0.84 (0.72, 0.98)* |
| Unknown | 0.76 (0.6, 0.96)* | 0.45 (0.34, 0.6)** |
| **Nipple areolar complex involvement^a^** |  |  |
| Yes | *Reference* | *Reference* |
| No | 0.76 (0.63, 0.91)** | 0.92 (0.75, 1.12) |
| Unknown | 0.57 (0.46, 0.71)** | 1.09 (0.84, 1.42) |
| **Estrogen receptor^a^** |  |  |
| Positive | *Reference* | *Reference* |
| Negative | 1.75 (1.55, 1.97)** | 0.87 (0.61, 1.23) |
| Unknown | 3.04 (1.45, 6.34)** | 436 (61.15, 3112.87)** |
| **Progesterone receptor^a^** |  |  |
| Positive | *Reference* | *Reference* |
| Negative | 1.77 (1.58, 1.98)** | 0.91 (0.73, 1.14) |
| Unknown | 2.85 (1.36, 5.96)** | 0.00 (0.00, 0.00)** |
| **HER2** |  |  |
| Positive | *Reference* | *Reference* |
| Negative | 0.77 (0.67, 0.87)** | 0.92 (0.77, 1.11)* |
| Unknown | 0.88 (0.61, 1.26) | 0.62 (0.39, 0.99) |
| **CK56** |  |  |
| Positive | *Reference* | *Reference* |
| Negative | 0.44 (0.38, 0.53)** | 0.77 (0.58, 1.02)* |
| Unknown | 1.00 (0.84, 1.18) | 1.93 (0.58, 6.4) |
| **EGFR** |  |  |
| Positive | *Reference* | *Reference* |
| Negative | 0.44 (0.38, 0.52)** | 0.68 (0.52, 0.9)** |
| Unknown | 1.04 (0.89, 1.21) | 0.56 (0.17, 1.83) |
| **Ki67 (%) categories** |  |  |
| 0-9 | *Reference* | *Reference* |
| 10'-19 | 1.69 (1.31, 2.19)** | 1 (0.76, 1.31) |
| 20'-29 | 2.28 (1.74, 2.99)** | 1.06 (0.78, 1.44) |
| 30'-39 | 2.65 (2.01, 3.50)** | 1.25 (0.92, 1.7) |
| 40'-49 | 2.49 (1.76, 3.54)** | 1.1 (0.75, 1.62) |
| 50'-59 | 3.55 (2.61, 4.82)** | 1.58 (1.1, 2.27)* |
| 60'-69 | 3.08 (2.19, 4.34)** | 1.32 (0.9, 1.93)* |
| 70'-79 | 3.01 (2.02, 4.51)** | 1.23 (0.8, 1.88) |
| 80'-89 | 3.70 (2.60, 5.27)** | 1.26 (0.84, 1.88) |
| 90'-100 | 3.94 (2.59, 6.00)** | 1.17 (0.7, 1.93) |
| Unknown | 2.96 (2.38, 3.68)** | 0.8 (0.63, 1.02) |
| **Radiotherapy categories** |  |  |
| Yes | *Reference* | *Reference* |
| No | 1.35 (1.19, 1.52)** | 1.39 (1.18, 1.63)** |
| Stop by patient | 43.36 (4.24, 443.17)* | 79.3 (30.63, 205.08)** |
| Unknown | 8.21 (4.75, 14.21)** | 4.72 (2.23, 10)** |
| **Chemotherapy categories^a^** |  |  |
| Yes | *Reference* | *Reference* |
| No | 0.54 (0.46, 0.62)** | 1.01 (0.83, 1.23) |
| Stop by patient | 1.93 (0.69, 5.44) | 0.6 (0.21, 1.72) |
| Unknown | 5.53 (2.5, 12.2)** | 0.88 (0.3, 2.55) |
| **Hormonal therapy categories** |  |  |
| Yes | *Reference* | *Reference* |
| No | 1.89 (1.68, 2.13)** | 1.52 (1.09, 2.13)* |
| Unknown | 6.13 (3.68, 10.2)** | 2.75 (1.26, 5.99)* |
| **Target therapy categories** |  |  |
| Yes | *Reference* | *Reference* |
| No | 1.20 (0.96, 1.50) | 1.48 (1.1, 1.99)** |
| Unknown | 0.18 (0.14, 0.23)** | 0.2 (0.15, 0.28)** |
| **Mammography categories** |  |  |
| C1 | *Reference* | *Reference* |
| C2 | 0.86 (0.13, 5.74) | 0.91 (0.26, 3.16) |
| C3 | 1.82 (0.27, 12.36) | 1.62 (0.46, 5.74) |
| C4a | 9.47 (1.36 ,66.19)* | 4.27 (1.08, 16.91)* |
| C4b | 28.00 (4.16, 188.62)** | 3.37 (0.84, 13.45) |
| C4c | 36.46 (5.39, 246.87)** | 5.77 (1.53, 21.81)** |
| C5 | 30.90 (4.36, 219.18)** | 3.71 (0.96, 14.3) |
| Unknown | 1.31 (0.20, 8.64)** | 1.44 (0.43, 4.86) |
| **Ultrasonography categories** |  |  |
| C1 | *Reference* | *Reference* |
| C2 | 1.07 (0.85, 1.34) | 1.02 (0.81, 1.29) |
| C3 | 2.01 (1.42, 2.85)** | 2.21 (1.57, 3.10)** |
| C4a | 17.15 (12.62, 23.31)** | 12.4 (8.71, 17.56)** |
| C4b | 84.36 (62.86, 113.22)** | 32.6 (21.27, 50.05)** |
| C4c | 124.73 (97.69, 159.25)** | 18.00 (11.77, 27.40)** |
| C5 | 71.13 (27.68, 182.80)** | 9.45 (4.72, 18.94)** |
| Unknown | 2.87 (2.49, 3.32)** | 1.93 (1.59, 2.33)** |
| **ALC (Absolute lymphocyte count)** | 0.66 (0.59, 0.75)** | 0.96 (0.82, 1.12) |
| **ANC (Absolute neutrophil count)** | 1.14 (1.11, 1.17)** | 1.24 (1.14, 1.34)** |
| **ALP (Alkaline phosphatase) ^b^** | 1.01 (1.00, 1.01)** | 1.00 (1.00-1.00) |
| **ALT (Alanine aminotransferse) ^b^** | 1.03 (0.91, 1.17) | 1.02 (0.79, 1.31) |
| **AST (Aspartate aminotransferase) ^a^** | 1.25 (1.04, 1.51)* | 0.86 (0.6, 1.24) |
| **Calcium^b^** | 0.82 (0.69, 0.98)** | 1.03 (0.84, 1.27) |
| **Total Cholesterol^a^** | 1.00 (1.00, 1.00)* | 1.00 (1.00-1.00) |
| **Glucose** | 1.00 (1.00, 1.01)** | 1.00 (1.00-1.01)* |
| **Hemoglobin** | 0.79 (0.74, 0.83)** | 0.87 (0.82, 0.93)** |
| **Total protein** | 0.67 (0.58, 0.79)** | 0.76 (0.63, 0.92)** |
| **WBC (White Blood Cell)** | 1.07 (1.06, 1.09)** | 0.90 (0.84, 0.98)** |
| **CEA (Carcinoembryonic antigen)** | 1.35 (0.98, 1.84)** | 1.00 (1.00-1.00)* |
| **CA 15-3** | 3.6 (3.23, 4.03)** | 3.25 (2.92, 3.63)** |

* p < 0.05, ** p < 0.01. a Prognostic feature selected by a backward selection procedure, b Prognostic feature selected from the clinical point of view. / Abbreviations: HR, hazard ratio; CI, confidence interval.

**Supplementary Table 2.** Performance of the final model in terms of model error (predicted – observed) over each of the 2/5/7 years. Patients were grouped by Pathologic T Stage. Median Absolute Error, Weighted Mean Absolute Error, and Maximum Error group were calculated at each group or bin.

| Prediction Time (year) | SubGroup | | N | Observed | Predicted | Model Error  (pred – obs) | wMAE |
| --- | --- | --- | --- | --- | --- | --- | --- |
| 2 | T1 | | 5,979 | 5.0% | 2.8% | -2.14% |  |
|  | T2 | | 3,740 | 9.0% | 5.9% | -3.05% | 2.59% |
|  | T3 | | 404 | 16.3% | 11.4% | -4.95% |  |
| 5 | T1 | | 3,587 | 12.5% | 10.2% | -2.34% |  |
|  | T2 | | 2,440 | 20.2% | 19.5% | -0.78% | 2.13% |
|  | T3 | | 252 | 33.7% | 32.9% | -0.79% |  |
| 7 | T1 | | 2,519 | 19.7% | 19.8% | 0.16% |  |
|  | T2 | | 1,859 | 28.5% | 31.6% | 3.17% | 1.90% |
|  | T3 | | 201 | 44.3% | 45.8% | 1.49% |  |
|  | | Median Absolute Error | | | 1.78% | |  |
|  | | Weighted Mean Absolute Error | | | 1.90% | |  |
|  | | Maximum Error | | | 4.95%  (T3 group at 2 year) | |  |

**Supplementary Table 3.** Performance of the final model in terms of model error (predicted – observed) over each of the 2/5/7 years. Patients were grouped by Pathologic N Stage. Median Absolute Error, Weighted Mean Absolute Error, and Maximum Error group were calculated at each group or bin.

| Prediction Time (year) | | SubGroup | N | Observed | Predicted | Model Error  (pred – obs) | wMAE |
| --- | --- | --- | --- | --- | --- | --- | --- |
| 2 | | N0 | 6,206 | 5.2% | 2.4% | -2.79% | 2.64% |
|  |  | N1 | 2,543 | 6.7% | 5.2% | -1.57% |  |
|  |  | N2 | 727 | 11.7% | 8.7% | -3.03% |  |
|  |  | N3 | 418 | 25.8% | 19.6% | -6.22% |  |
| 5 | | N0 | 3,741 | 12.2% | 9.4% | -2.75% | 2.29% |
|  |  | N1 | 1,684 | 16.9% | 16.4% | -0.48% |  |
|  |  | N2 | 506 | 26.9% | 27.5% | 0.59% |  |
|  |  | N3 | 306 | 46.4% | 48.7% | 2.29% |  |
| 7 | | N0 | 2,671 | 18.8% | 18.5% | -0.30% | 2.15% |
|  |  | N1 | 1,249 | 24.5% | 26.7% | 2.24% |  |
|  |  | N2 | 395 | 36.5% | 43.8% | 7.34% |  |
|  |  | N3 | 252 | 58.7% | 66.7% | 7.94% |  |
|  | Median Absolute Error | | | | 3.05% | |  |
|  | Weighted Mean Absolute Error | | | | 2.15% | |  |
|  | Maximum Error | | | | 7.94%  (N3 group at 7 year) | |  |

**Supplementary Table 4.** Performance of the final model in terms of model error (predicted – observed) over each of the 2/5/7 years. Patients were grouped by ER/PR/HER2 Status. Median Absolute Error, Weighted Mean Absolute Error, and Maximum Error group were calculated at each group or bin.

| Prediction Time (year) | SubGroup | N | Observed | Predicted | Model Error  (pred – obs) | wMAE |
| --- | --- | --- | --- | --- | --- | --- |
| 2 | ER and PR-/HER2+ | 1,015 | 8.5% | 6.9% | -1.58% | 2.65% |
|  | ER and PR-/HER2- | 1,515 | 12.5% | 7.3% | -5.28% |  |
|  | ER or PR+/HER2+ | 1,187 | 8.8% | 6.6% | -2.19% |  |
|  | ER or PR+/HER2- | 6,239 | 5.1% | 2.8% | -2.28% |  |
| 5 | ER and PR-/HER2+ | 682 | 17.9% | 18.3% | 0.44% | 2.27% |
|  | ER and PR-/HER2- | 1,053 | 23.4% | 19.9% | -3.42% |  |
|  | ER or PR+/HER2+ | 755 | 19.5% | 20.3% | 0.79% |  |
|  | ER or PR+/HER2- | 3,676 | 13.7% | 11.8% | -1.93% |  |
| 7 | ER and PR-/HER2+ | 504 | 25.4% | 28.6% | 3.17% | 2.00% |
|  | ER and PR-/HER2- | 826 | 31.5% | 32.4% | 0.97% |  |
|  | ER or PR+/HER2+ | 567 | 27.3% | 30.3% | 3.00% |  |
|  | ER or PR+/HER2- | 2,581 | 21.8% | 22.7% | 0.97% |  |
|  | Median Absolute Error | | | 1.86% | |  |
|  | Weighted Mean Absolute Error | | | 2.00% | |  |
|  | Maximum Error | | | 5.28%  (ER and PR-/HER2- group at 2 year) | |  |

**Supplementary Table 5.** Performance of the final model in terms of model error (predicted – observed) over each of the 2/5/7 years. Patients were grouped by EGFR Status. Median Absolute Error, Weighted Mean Absolute Error, and Maximum Error group were calculated at each group or bin.

| Prediction Time (year) | | SubGroup | N | Observed | Predicted | Model Error  (pred – obs) | wMAE |
| --- | --- | --- | --- | --- | --- | --- | --- |
| 2 | | EGFR+ | 1,658 | 11.0% | 6.8% | -4.16% | 2.54% |
|  |  | EGFR- | 5,769 | 4.5% | 2.4% | -2.08% |  |
| 5 | | EGFR+ | 943 | 24.2% | 21.6% | -2.55% | 2.40% |
|  |  | EGFR- | 2,774 | 14.2% | 12.1% | -2.16% |  |
| 7 | | EGFR+ | 594 | 39.7% | 42.3% | 2.53% | 2.09% |
|  |  | EGFR- | 1,607 | 26.3% | 27.3% | 1.06% |  |
|  | Median Absolute Error | | | | 2.42% | |  |
|  | Weighted Mean Absolute Error | | | | 2.09% | |  |
|  | Maximum Error | | | | 4.16%  (EGFR+ group at 2 year) | |  |

**Supplementary Table 6.** Performance of the final model in terms of model error (predicted – observed) over each of the 2/5/7 years. Patients were grouped by CK56 Status. Median Absolute Error, Weighted Mean Absolute Error, and Maximum Error group were calculated at each group or bin.

| Prediction Time (year) | | SubGroup | N | Observed | Predicted | Model Error  (pred – obs) | wMAE |
| --- | --- | --- | --- | --- | --- | --- | --- |
| 2 | | CK56+ | 1,255 | 12.0% | 7.1% | -4.86% | 2.54% |
|  |  | CK56- | 6,174 | 4.7% | 2.6% | -2.07% |  |
| 5 | | CK56+ | 719 | 25.2% | 22.1% | -3.06% | 2.40% |
|  |  | CK56- | 2,999 | 14.7% | 12.7% | -2.07% |  |
| 7 | | CK56+ | 465 | 40.2% | 43.2% | 3.01% | 2.09% |
|  |  | CK56- | 1,737 | 27.1% | 28.2% | 1.04% |  |
|  | Median Absolute Error | | | | 2.68% | |  |
|  | Weighted Mean Absolute Error | | | | 2.09% | |  |
|  | Maximum Error | | | | 4.86%  (CK56+ group at 2 year) | |  |
